# Elite Status‐Seeking and Class Reproduction in Civil Society: An Analysis of Corporate Elite Appointments to Charity Boards

**DOI:** 10.1111/1468-4446.13201

**Published:** 2025-02-27

**Authors:** Tom Mills, Narzanin Massoumi

**Affiliations:** ^1^ Department of Society and Politics Aston University Birmingham UK; ^2^ Department of Social and Political Sciences, Philosophy and Anthropology University of Exeter Exeter UK

**Keywords:** charities, civil society, class reproduction, computational social science, corporate elites

## Abstract

This article examines the relationship between economic elites and civil society by analysing the appointments of corporate elites to the boards of charitable companies in the UK. Whilst previous research has usually focused on who among the corporate elite hold positions in key civil society organisations, and the extent to which these organisations are integrated into corporate networks, we use data on the nature and operations of civil society organisations to identify which are more likely to attract corporate elites as board members. Using a dataset of over thirty‐one thousand UK incorporated companies registered with the Charity Commission of England and Wales, we examine the appointments of corporate elite to these organisations over a 10‐year period. Based on these appointments, we are able to offer insights into the social networks, values and interests of the corporate elite as a whole. We find that the UK corporate elite are more likely to join the boards of larger, high‐status charities, and those that support traditional upper‐class culture and class reproduction. We also find they are relatively more likely to join organisations that seek to shape politics and society—such as foundations distributing grants, or think tanks undertaking public policy research and advocacy—than those involved in the provision of welfare and social services. Taken together, the findings are suggestive of a status‐seeking, culturally highbrow and secular economic elite, that is more traditional than meritocratic, and more concerned with shaping policy and supporting the institutions of their class, than directly supporting disadvantaged groups.

## 1 Introduction

This article examines the relationship between economic elites and civil society by analysing the appointments of corporate elites to the boards of charitable companies in the UK. Existing research has typically explored this question from the perspective of the corporate elite, focusing on who among them holds positions in key civil society organisations, as well as the extent to which such organisations are integrated into corporate networks. In contrast, this study investigates the characteristics of civil society organisations to identify which are more likely to attract corporate elite members to their boards. In doing so it provides insights into the social networks, values and interests of the corporate elite. Drawing on a dataset of 31,666 charities, it argues that corporate elite engagement in civil society is less about tackling systemic inequalities and more about consolidating their social power, legitimising their dominance, and supporting the institutions and cultural values that sustain and reproduce their class.

The article begins with a review of the existing literature on elite engagement in civil society, situating the study within debates on class power and status, and differentiating our work from research on elite and corporate philanthropy or corporate social responsibility. It then outlines the data and methods used, including the construction of a unique dataset and the operationalisation of key variables. The results section presents both bivariate and multivariate analyses of corporate elite appointments to charity boards, identifying distinct patterns of engagement. Finally, the conclusion reflects on the implications of these findings for understanding the social values and interests of the UK corporate elite.

This study is part of a tradition of politically engaged empirical research that, building on C. Wright Mills's seminal text (C. W. Mills 2000), identifies and analyses powerful groups in society. Such research has often employed social network analysis, with work on corporate elites focussing particularly on the integrative role played by directors on the board of two or more large companies (Sapinski and Carroll 2018). Some classic network‐based studies of corporate elites also analysed extra‐corporate appointments (Domhoff 1967; Useem 1984) and there is a growing body of more recent work on corporate elites integrating such appointments and analysing the extent to which large companies are connected to large organisations in other social sectors via shared directorships (e.g. Denord Lagneau‐Ymonet, and Thine 2018; Huijzer and Heemskerk 2021; Rossier et al. 2022). This has long been a focus in power structure research (Denord Lagneau‐Ymonet, and Laurens 2020; Domhoff 1980, 2017b), which has examined not just networks of large corporations, but also leading foundations, think‐tanks and policy planning groups which facilitate social and policy cohesion among corporate elites (Burris 2005; Domhoff 2017a; T. Mills and Domhoff 2023; Salzman and Domhoff 1983).

Integrating civil society organisations into elites research requires acknowledging the contradictory role such organisations can play. As Domhoff (2009) argues, whilst some organisations genuinely challenge corporate power, many not‐for‐profits act to ameliorate social conflicts. Others are overtly reactionary, seeking to overturn political gains by progressives, backed by the more conservative members of the corporate community. Carroll and Sapinski (2018) similarly suggest that the not‐for‐profit sector is the section of society most independent from corporate power, but note that the corporate elite are well represented on the boards of foundations, hospitals and universities. Such organisations, they argue, play an important role in the exercise of capitalist hegemony (Carroll and Sapinski 2017).

A few studies of corporate elites have focused on civil society connections in particular. Moore et al. (2002) examine the interpenetration of the third sector by state and private sector elites using a dataset of directors or trustees of the largest companies, charities and foundations in the US, as well as individuals serving on federal advisory committees. Whilst charities and foundations were much less central in the resulting network than corporations, around half of the top charities shared directors with another top institution. Vidovich and Currie (2012), meanwhile, suggest a closer relationship between corporate and ‘third sector’ elites in Western Australia, identifying an ‘inner circle’ of directors straddling the two sectors. A more recent study by Gulbrandsen (2020) also contrasts to some extent with Moore et al. (2002), finding that individuals involved in voluntary organisations in Norway are among the most connected of the country's business elite and serve as ‘bridging actors’ facilitating elite integration across sectors.

There is a growing body of research adopting similar methods to examine power dynamics within civil society, notably work examining board interlocks among not‐for‐profit organisations in the US (for an overview and discussion see Yoon 2020). Messamore (2021), for example, shows a growth of interlocking directorates between nonprofit community‐based organisations, which he argues is suggestive of an emerging integrated ‘civic elite’. Such research, though, is mainly concerned with the diffusion of information and governance practices among not‐for‐profit organisations and/or the relationship between inter‐organisational connections and grant capture (Bloch Harris, and Peterson 2020; Esparza and Jeon 2013; Faulk, McGinnis Johnson, Lecy, et al. 2017; Faulk, et al. 2016; Paarlberg Hannibal, and McGinnis Johnson 2020). How the sector connects with the corporate world, or how it relates to the broader societal power structure is not a central concern (although see Gulbrandsen 2020). Additionally, there is a growing body of work on civil society elites in Europe (Johansson and Meeuwisse 2024a; Johansson and Uhlin 2020; O'Brien Rees, and Taylor 2022), but as Johansson and Meeuwisse (2024b) note, the focus of this work is similarly on elites of civil society, as opposed to elites in civil society, which is the focus here.

## 2 Why do Corporations and Corporate Elites Engage With Civil Society?

Whilst much existing work on civil society in elite studies has focused on intra‐elite integration, other studies have examined involvement in civil society organisations as a mechanism for elite legitimation and social action. Extensive research across a range of disciplines has examined how elite and corporate philanthropy, Corporate Social Responsibility (CSR) and what has been called ‘philanthrocapitalism’, advance the interests of corporations and the wealthy beyond the economic sphere into politics and civil society (for reviews see Barman 2017; Cha and Rajadhyaksha 2021; Gautier and Pache 2015; Haydon Jung, and Russell 2021).^1^ Research within business ethics, marketing and management studies has identified CSR and corporate philanthropy as means through which large companies address the social and environmental concerns of ‘stakeholders’. In this research, civil society engagement is analysed from the organisational perspective, and can be understood as a way of enhancing a firm's public reputation—which is particularly important in the case of consumer‐orientated businesses (Brammer and Millington 2006; Campbell and Slack 2006)—addressing the concerns and expectations of consumers, employees and potential employees, shaping modes of civic engagement in ways amenable to corporate interests, managing political risk and co‐opting oppositional social movements (Banerjee 2008; Costas and Kärreman 2013; Hanlon and Fleming 2009).

Sociological studies, meanwhile, have examined how economic elites engage with civil society organisations as a mechanism to enhance their class power, status and legitimacy. Analysed from this perspective, engagement in the sector may provide opportunities to: consolidate and expand social networks (Maclean Harvey, and Kling 2017; Ostrower 2020); support institutions sustaining upper class culture and facilitating class reproduction (Glucksberg and Russell‐Prywata 2020; Odendahl 1990; Ostrower 1997, 2020); attain broader social status in an effort to legitimise their wealth and power (Adloff 2016; Maclean Harvey, and Kling 2017; McGoey 2015); and (equally from the perspective of the company) shape public policy and knowledge production (Bertrand et al. 2020; Callahan 2017; Fisher 1983; Fooks et al. 2013; Goss 2016; Page Seawright, and Lacombe 2018).

These two perspectives—the organisational on the one hand and the elites/class perspective on the other—arise from different analytical frameworks associated with different disciplines. But as Scott (1991a) and Sapinski and Carroll (2018) have argued, power in modern capitalist societies can be fruitfully analysed from both an organisational perspective, where the relevant actors are firms, or an interpersonal perspective where the relevant actors are members of a social class or elite group. Whilst cognisant of both, this article draws on and extends the class and elites based sociological dimension. It examines the appointments of the directors of the largest UK companies to the boards of a subset of civil society organisations in the UK: charitable companies in England and Wales, meaning organisations incorporated under the Companies Act and registered with the Charity Commission of England and Wales. We provide further details on this subset of organisations in the data and methods section below and in Appendix A.

We use the term civil society to refer to the sector of capitalist society that is distinct from both the state and the market (Roginsky and Shortall 2009), which, whilst not a core part of capitalist power structures, we consider an important sphere for researching elites and power. We consider civil society as broader than the charitable sector, but use corporate elite appointments to charitable companies for the purpose of empirical investigation. We discuss this further, and note some limitations of this approach in Appendix A.

The corporate elite (which we define below) is a hard‐to‐reach group, but they leave behind digital evidence of their activities, which we use to our advantage (Halford and Savage 2017). We make use of administrative data that allows appointments to be assessed systematically, over time and at scale. We adopt what has been termed a ‘symphonic’ approach to social science, integrating diverse data sets and statistical analysis with theoretical understanding (Halford and Savage 2017). Our approach responds to calls for sociologists to make use of datasets now routinely produced by public and private bodies (Burrows and Savage 2014; Savage 2009; Savage and Burrows 2007), data that it has been suggested is particularly useful for work on elites (Burrows 2015; Khan 2012).

## 3 Expected Patterns of Corporate Elite Civil Society Engagement

As noted above, this study seeks to identify which types of civil society organisations are more likely to attract members of the corporate elite to their boards. If the UK corporate elite share a broad set of social values based in their shared economic interests, as is detailed in existing studies (Boswell and Peters 1997; Davis 2019; Lazar 2016), then this should give rise to distinct patterns of engagement. If, on the contrary, they do not differ much from charity trustees in general, then we would expect to find no statistically significant patterns emerge from their distribution among the boards of different types of organisations.

Of course, we can be more specific. The corporate elite is a group that is used to exercising considerable power, not just within the organisations they lead, but more broadly. We expect therefore that they will be more attracted to influential and well‐resourced charities than to smaller charities. We also expect, for the same reason, that they will be more attracted to organisations that seek to shape politics and society—for example to foundations distributing grants, or think tanks undertaking public policy research and advocacy—than those involved in the provision of welfare and social services. The UK corporate elite is international in composition and orientation (Cronin 2012; Hartmann 2017), and we expect therefore that they would be more attracted to the boards of charities with international operations, than those with national or regional activities.

A particular focus of existing research on corporate elites has been the extent to which they are drawn from upper‐class backgrounds, and/or from ‘elite’ educational institutions (Buck 2018; Flemmen 2012; Lee et al. 2021; Maclean Harvey, and Press 2006; Mastekaasa 2004; Stanworth and Giddens 1974). In older literature on the UK corporate elite in particular, shared class backgrounds and ‘establishment’ institutions—especially public schools, ‘Oxbridge’ and London social clubs—have been assumed to facilitate social and policy cohesion among this group (Bond 2007; Scott 1991b; Whitley 1973). More recent literature has suggested that financialisaton and globalisation have eroded these traditional mechanisms of class solidarity and elite cohesion (Davis 2018; Feldmann and Morgan 2021; Sampson 2005; Savage 2015; Wilks 2013). Whilst this study does not address questions of social origin, we are interested in the extent to which, given such debates, the corporate elite in the UK remain supportive of traditional institutions of intergenerational class reproduction. We address this by examining tendency of the corporate elite to join the boards of charities associated with private schools and with Oxford and Cambridge universities.

Alongside these more instrumental motives, the literature on elites and CSR leads us to expect that the corporate elite may also seek through civil society to justify and legitimise their wealth and power. Such legitimation strategies, if present, may take a more or less meritocratic or traditional character, strategies that exist in tension with one another. Research on the social attitudes of the wealthy has consistently found that their wealth is justified by appeals to meritocracy, meaning it is understood to be deserved on the basis of their natural talent and/or effort (e.g. Atria et al. 2020; Kantola and Kuusela 2018, Khan 2011; Savage 2015). This is more marked in less equal societies like the UK (Friedman et al. 2023), a finding Mijs (2021) has described as a ‘paradox of inequality’. Research finds some variation between countries and groups. For example, Atria et al. (2020) find a greater emphasis on individual talent among economic elites in Chile; wealthy Finnish entrepreneurs emphasise hard‐work and risk‐taking (Kantola and Kuusela 2018); whilst Schimpfössl (2024) finds that Russian multi‐millionaires and billionaires appeal to both talent and hard‐work, but are also more likely than the wealthy elsewhere to understand talent as being based in genetics. In the UK specifically, research has found that financial elites appeal to the market as a legitimate measure of an individual's social contribution, specifically in relation to performance‐related pay (Hecht 2022), while UK elites more generally emphasise talent more than hard work, in contrast to elites in Denmark (Friedman et al. 2023).

Given the strong emphasis on talent‐based meritocracy, we would expect that the UK corporate elite will be more likely to be involved with charities that reflect and promote meritocratic ideology. To be clear, the expectation is not that appointments will reveal a genuine commitment to equality (and in any case we are not able to measure individual motives), but rather a tendency to support organisations that promote what Littler (2017) has called ‘myths of mobility’. This is difficult to capture with the available data (detailed further below), but we would expect to see a greater level of involvement in charities focussing on education and young people, since early life and educational disadvantage are conspicuous obstacles to meritocracy. We might also expect an association with charities involved in sport, which whilst highly unequal, has nevertheless often been seen as a rules‐based ‘level playing field’ and as a site of social mobility for talented individuals who excel in a competitive environment (LaVaque‐Manty 2009; Spaaij Farquharson, and Marjoribanks. 2015).

Meritocracy ideology, though, is just one way in which social inequalities can be justified. Historical and sociological research has detailed how economic inequalities have been legitimised symbolically with appeals to culture and tradition (real and imagined) (Bourdieu 1986; DiMaggio 1982; Hobsbawm and Ranger 2012; Levine 1990; Ostrower 2020; Smith 2023; Wu 2003). Research in cultural sociology, meanwhile, has detailed a shift among the affluent away from traditional, highbrow pursuits, towards a greater embrace of popular culture and ‘omnivorous’ cultural consumption, more in keeping with a meritocratic ethos (Bennett et al. 2009; Chan 2019; Flemmen Jarness, and Rosenlund 2018; Friedman and Reeves 2020). We are interested, therefore, in the extent to which corporate elites may still seek legitimation via involvement with older, high status, ‘establishment’ charities, and/or those involved in heritage, and the sorts of ‘highbrow’ cultural activities associated with the traditional upper classes (e.g. opera, ballet, etc).

## 4 Research Questions and Hypotheses

In summary, we examine the distribution of corporate elites among the boards of different types of civil society organisations over a 10‐year period, taking this as evidence of their social networks, values and interests. We address the following research questions:

- Are the appointments of corporate elites to the board of charities suggestive of distinct networks, values and interests?
- Which categories of charities are more likely to attract corporate elites to their boards?
- Are corporate elites more likely to join larger, high status charities?
- Are corporate elites more likely to join charities involved in traditional ‘highbrow’ culture?
- Are corporate elites more likely to join organisations associated with meritocratic values?
- Are corporate elites more likely to join organisations involved in shaping policy and knowledge production?
- Are corporate elites more likely to join charities supporting intergenerational class reproduction?

We expect corporate elite engagement with civil society to be driven by motives related to status‐seeking, social legitimation and the consolidation of social power. Based on these assumptions, we hypothesise that the corporate elite in the UK are more likely to join the boards of charities that support traditional ruling‐class culture and facilitate class reproduction. Additionally, we anticipate that their involvement will be particularly pronounced in charities that shape policy and knowledge production, such as think tanks and grant‐distributing foundations. Given the influence and resources typically associated with the corporate elite, we expect them to gravitate toward influential, well‐resourced and high‐status charities rather than those primarily focused on providing welfare and social services. Further, we hypothesise that their engagement will reflect a preference for organisations that promote meritocratic values, particularly those centred on education, youth, and sports. Finally, we expect to find a significant association between the corporate elite and charities involved in heritage preservation and highbrow cultural activities, such as classical arts, which are traditionally associated with the upper classes. The operationalisation of these hypotheses in relation to our dataset is detailed in the following section.

## 5 Data and Methods

### 5.1 Identifying the UK Corporate Elite

We adopt a positional method of elite identification (Hoffmann‐Lange 2007) and follow Bukodi and Goldthorpe (2021, 673) in understanding elites as ‘“small‐N entities”, clearly distinct from social classes’. Whilst elites are identifiable persons occupying specific social positions, classes are larger aggregations grouped around common economic situations (Giddens 1974; Scott 2003). We therefore consider capitalist classes and economic elites to be distinct, although sometimes overlapping, analytical categories. Our focus in this study is on a subset of the latter group: the UK corporate elite, which is the equivalent of what Domhoff in the US has called the ‘corporate leadership group’ (Domhoff 2022; T. Mills and Domhoff 2023). This relatively small group is defined according to the legal and managerial authority it exercises within the UK's largest companies. This definition does not include major shareholders, who may exercise ultimate control, but whom we consider a distinct social group defined according to ownership rather than organisational authority. As such, we follow the distinction in law and corporate governance between management and ownership, which is also an important analytical distinction in the sociological literature (Giddens 1974; Scott 2003).

We identify the UK corporate elite first by compiling a list of the largest UK companies. Research on corporate elites has usually been based on the largest companies in a sample year, often including both financial and non‐financial firms. Sample sizes vary, but studies usually include 200 to 500 of the largest companies in a particular country (for a summary of sampling criteria used see Brullebaut et al. 2022; Huijzer and Heemskerk 2021). Huijzer and Heemskerk (2021) conclude that in network studies, samples of 250 (or more) companies produce reliable results and, importantly for our purposes, capture the bulk of extra‐corporate appointments.

We identified the 500 largest companies in the UK by turnover in every year from 2013 to 2022 using the business directory Fame. In each sample year, Fame lists some companies within the top 500 companies from the same corporate group that report the same consolidated group accounts (e.g. Rolls‐Royce plc and Rolls‐Royce Holdings plc). In these cases, we include both legal entities, but expand the total companies included in that year to account for the duplicated group. Thus, in every sample year we have 500 corporate groups, but a slightly larger number of legal entities. Finally, to allow for any short‐term anomalies, we exclude companies appearing in only one sample year. In total this yielded 830 companies. Using the registration numbers of these companies, we queried the API of the official UK company registry, Companies House, to identify every director in every year that a company appeared in a top 500 list. This is our UK corporate elite, a total of 9035 individuals who in our sample period have served as a director of one of the 500 largest UK companies, each of whom is associated with a unique Companies House appointments ID.

### 5.2 Dataset and Variables

Whilst the UK corporate elite is the starting point for this study, it is not the unit of analysis. Rather this is charitable companies, a subset of charities active at the time of data extraction which are also incorporated under the Companies Act 2006 and therefore registered at Companies House. After dropping a small number of organisations recording invalid company registration numbers, or which had not provided financial data at the time of data extraction, this yielded at total of 31,666 charitable companies. We provide further details on the organisations included in our analysis in Appendix A.

Our combined use of Charity Commission and Companies House data has some limitations, which we discuss below, but two crucial advantages. First, whilst Companies House provides some data on the activities of companies, such as basic accounting data and a classification of activities (Standard Industrial Classification [SIC] codes), the Charity Commission publishes much more extensive structured data on the nature and operations of organisations. Second, using Companies House appointments data allows us to link the corporate elite to charities with a level of accuracy that would not otherwise be possible. Central to our analysis here is the presence of any member of the UK corporate elite, as defined above, on the board of these organisations at any point in our sample period. This is our outcome variable, which we construct using Companies House IDs, an approach that strongly favours *precision* over *recall*, or put another way, data quality over data quantity. Third, using Companies House data allows us to capture a longer time period than would otherwise have been possible, since only current trustees are recorded in Charity Commission data, whilst Companies House records former directorships.

We enrich the Charity Commission data with data from three other sources, which allow us to more effectively address our research questions. Our royal patronage variable is used as a measure of status and prestige, and draws on research conducted by the charity research consultancy, Giving Evidence (Fiennes and McLain 2020). That research identified organisations with a member of the British monarchy as a patron by scraping the official website of the British monarchy, as well as the official websites of the Prince of Wales (now King Charles), the Duchess of Cornwall (now Queen Camilla), the Duke of York (Prince Andrew) and the Duchess of Cambridge (Kate Middleton). This identified a total of 1187 charities with a royal patron, which are then identified in our dataset by their Charity Commission organisation number.

To assess the extent to which corporate elites are attracted to organisations involved in policy and knowledge production, we identify think tanks among the charitable companies. To do so we made use of an open dataset compiled by the organisation, On Think Tanks. Like the Charity Commission data, this dataset includes the organisations' websites, allowing us to easily identify think tanks among the charitable companies. Of the 162 UK think tanks, we identified 59 among the charitable companies in our data.

To examine class reproduction, we identify charities associated with private schools or with ‘Oxbridge’. In the case of our private schools variable, we include not only private schools, but also associated charities such as alumni societies and parent teacher associations. To do so we use data on ‘independent’ schools published by the UK Department of Education, matching these schools to the charities via their websites and postcodes. Any charity sharing a website with a DoE registered private school was categorised as such, as was any charity with ‘school’ in the charity name that shared an exact postcode with a private school. We identified ‘Oxbridge’ charities on the basis that a charity website uses the official domain of either Oxford or Cambridge universities.

The remaining predictor variables we use in our analysis are based solely on data obtained from the Charity Commission. These are for the most part single observations, meaning we are not able to observe changes during our sample period,^2^ and each required some transformation to make them amenable to analysis. In the case of charities' activities, we produce discrete binary variables based on whether a particular category of activity is reported, and in a few cases we combine the Charity Commission's categories. In total we produce 22 variables based on the Charity Commission's classifications.

Our charity age variable is based on the date of registration recorded in the register and the end of our sample period. We then produce an ordinal variable with three categories: 0–20, 21–40 and 41+ years. Our variable for charity size is based on the mean annual gross income reported in the Charity Commission's data files, which we assign to three quantiles.

In the case of a charity's areas of operations, organisations can report multiple continents, countries, UK nations, and regions. We create from this a simplified ordinal variable with four categories. Charities categorised as ‘local’ are those with operations in specific regions of any of the UK nations, whilst those categorised as ‘national’ have operations either across the UK or across one of its constituent nations. Charities we categorise as ‘international’ are those with some operations outside of the UK and those we categorise as ‘global’ are those that operate on more than one continent.

Finally, we identify charities involved in ‘highbrow’ culture (Levine 1990; Reeves 2019) using a list of strings denoting such activities (namely: theatre, museum, gallery, opera, ballet, classical, symphony, philharmonic), checking if any of these terms appeared in a charity name or activities.

In addition to the data detailed above, the Charity Commission provides a range of other data on registered charities that are not operationalised in this research. These include some administrative and financial data that are not of theoretical interest, as well as detailed financial data available only for charities with an annual income over £500,000. The Charity Commission variables not included in our analysis are listed in the Appendix in Table A2, along with counts and percentages for the missing values for each for organisations included in our analysis. Detailed guidance on the data provided by the Charity Commission is available online (Charity Commission 2024), and the original Charity Commission data, and the Python scripts used to construct each of the above variables, and to produce the final dataset, are available on GitHub.^3^

## 6 Results and Discussion

### 6.1 Bivariate Analysis

A total of 819 of the 31,666 charitable companies in our dataset (2.59%) have, or have had, a member of the corporate elite on their board. The number of charitable companies in each category of our predictor variables are reported in Table 1. To make the results in this table more readable, there are different subsections of the table: first the five binary variables we constructed that are of particular theoretical interest; then our three ordinal variables; then two sections together containing binary variables based on six Charity Commission classifications of primary theoretical interest and finally the remaining 16 Charity Commission classifications.

**TABLE 1:** Count, percentage and odds ratio for charitable companies with a member of the corporate elite on the board, by predictor variables.

			Corporate elite trustee		
		*N*	Yes (%)	No (%)	Odds ratio
Royal patronage^**^	Yes	404	63 (15.59)	341 (84.41)	7.45
	No	31,262	756 (2.42)	30,506 (97.58)	
High culture^**^	Yes	960	43 (4.48)	917 (95.52)	1.81
	No	30,706	776 (2.53)	29,930 (97.47)	
Private school^**^	Yes	720	82 (11.39)	638 (88.61)	5.27
	No	30,946	737 (2.38)	30,209 (97.62)	
Oxbridge	Yes	32	0 (0.00)	32 (100.00)	0.00
	No	31,634	819 (2.59)	30,815 (97.41)	
Think tank^**^	Yes	59	16 (27.12)	43 (72.88)	14.27
	No	31,607	803 (2.54)	30,804 (97.46)	
Size^**^	Large	10,587	634 (5.99)	9953 (94.01)	9.36
	Medium	10,580	114 (1.08)	10,466 (98.92)	1.60
	Small	10,499	71 (0.68)	10,428 (99.32)	
Age^**^	41+	2680	155 (5.78)	2525 (94.22)	2.81
	21–40	8196	220 (2.68)	7976 (97.32)	1.26
	0–20	20,790	444 (2.14)	20,346 (97.86)	
Area of operations^**^	Global	1995	96 (4.81)	1899 (95.19)	2.33
	International	2797	82 (2.93)	2715 (97.07)	1.39
	National	5464	187 (3.42)	5277 (96.58)	1.64
	Local	21,410	454 (2.12)	20,956 (97.88)	
Legitimation—traditional					
Armed forces/emergency services^**^	Yes	187	12 (6.42)	175 (93.58)	2.61
	No	31,479	807 (2.56)	30,672 (97.44)	
Arts/culture/heritage/science^*^	Yes	7088	203 (2.86)	6885 (97.14)	1.15
	No	24,578	616 (2.51)	23,962 (97.49)	
Environment/conservation/heritage^**^	Yes	4590	154 (3.36)	4436 (96.64)	1.38
	No	27,076	665 (2.46)	26,411 (97.54)	
Legitimation—meritocratic					
Amateur sport^**^	Yes	4105	80 (1.95)	4025 (98.05)	0.72
	No	27,561	739 (2.68)	26,822 (97.32)	
Education/training^**^	Yes	20,408	586 (2.87)	19,822 (97.13)	1.40
	No	11,258	233 (2.07)	11,025 (97.93)	
Young people	Yes	17,242	457 (2.65)	16,785 (97.35)	1.06
	No	14,424	362 (2.51)	14,062 (97.49)	
Other charity commission variables					
Accommodation/housing	Yes	2580	63 (2.44)	2517 (97.56)	0.94
	No	29,086	756 (2.60)	28,330 (97.40)	
Advocacy/advice/info	Yes	14,271	353 (2.47)	13,918 (97.53)	0.92
	No	17,395	466 (2.68)	16,929 (97.32)	
Animals	Yes	839	16 (1.91)	823 (98.09)	0.73
	No	30,827	803 (2.60)	30,024 (97.40)	
Disability^**^	Yes	11,255	239 (2.12)	11,016 (97.88)	0.74
	No	20,411	580 (2.84)	19,831 (97.16)	
Economic/community development/employment	Yes	7007	163 (2.33)	6844 (97.67)	0.87
	No	24,659	656 (2.66)	24,003 (97.34)	
Ethnic group^**^	Yes	4319	68 (1.57)	4251 (98.43)	0.57
	No	27,347	751 (2.75)	26,596 (97.25)	
Health/saving lives	Yes	7808	207 (2.65)	7601 (97.35)	1.03
	No	23,858	612 (2.57)	23,246 (97.43)	
Human rights/equality/diversity^**^	Yes	1757	28 (1.59)	1729 (98.41)	0.60
	No	29,909	791 (2.64)	29,118 (97.36)	
Makes grants^**^	Yes	8449	358 (4.24)	8091 (95.76)	2.18
	No	23,217	461 (1.99)	22,756 (98.01)	
Older people^**^	Yes	9317	171 (1.84)	9146 (98.16)	0.63
	No	22,349	648 (2.90)	21,701 (97.10)	
Overseas aid	Yes	1963	46 (2.34)	1917 (97.66)	0.90
	No	29,703	773 (2.60)	28,930 (97.40)	
Poverty^**^	Yes	8329	184 (2.21)	8145 (97.79)	0.81
	No	23,337	635 (2.72)	22,702 (97.28)	
Public/mankind	Yes	19,155	480 (2.51)	18,675 (97.49)	0.92
	No	12,511	339 (2.71)	12,172 (97.29)	
Recreation^**^	Yes	3084	53 (1.72)	3031 (98.28)	0.63
	No	28,582	766 (2.68)	27,816 (97.32)	
Religion^**^	Yes	5626	42 (0.75)	5584 (99.25)	0.24
	No	26,040	777 (2.98)	25,263 (97.02)	
Research^**^	Yes	5085	245 (4.82)	4840 (95.18)	2.29
	No	26,581	574 (2.16)	26,007 (97.84)	

*N* = 31,666.

^*^ Significant at the 5% level.

^**^ Significant at the 1% level.

In Table 1 we also present the cross‐tabulations for our outcome variable and each of these predictor variables, as well as the odds ratios. The odds ratios identify the extent to which each category increases or decreases the likelihood that a corporate elite will be on the board of an organisation. For example, a charitable company with royal patronage is more than seven times more likely to have a corporate elite on the board than a charitable company with no royal patronage. Those figures below 1.0 indicate that a corporate elite is less likely to be on the board. In the case of the ordinal variables, the odds are based on the lowest category. So, for example, a large charitable company is more than nine times more likely to have a corporate elite on its board compared to a small charitable company.

Note that whilst charitable companies can be regarded as a (non‐probability) sample of a broader population of charities, we are not making statistical inferences to a broader population (we discuss this in more detail in Appendix A). For this reason, conventional measures of statistical significance do not apply. Nevertheless, we report statistical significance as a measure of confidence that the observed distribution of corporate elites among different categories of organisations occurred as a result of an underlying social mechanism, rather than by chance. We assess this with a simple permutation test, a non‐parametric method conceptually similar to ‘bootstrapping’, except that rather than producing a new sample by resampling with replacement in order to estimate sample variability, resampling without replacement is used for hypothesis testing. In experimental research design this would classically involve repeatedly randomly reassigning cases to a test and control group, and the relevant test statistic (e.g. difference in means) being recorded for every iteration (see Collingridge 2013). We similarly randomly reassign our outcome variable—shuffling it essentially—among the charities 10,000 times and with each iteration record if the simulated result for each predictor variable is equal to or more extreme than the observed value. This yields a proportion that can be used for hypothesis testing in the same way as a *p*‐value in inferential statistics. We report the level at which the results for our variables are statistically significant under this test (at the 5% and 1% levels) with asterisks in Table 1.

We can note a few things at this stage. First, there are no corporate elites on the boards of any of the 31 ‘Oxbridge’ charities. Whilst this may seem to suggest little relationship with these institutions of class reproduction, the finding is not surprising given the very small number of organisations, and as you would expect it is not statistically significant (the same result was obtained in 44% of the iterations of the permutation test). The association with private school charities, meanwhile, our other main indicator of involvement in organisations of class reproduction, is striking. A member of the corporate elite has served on the board of 82 of the 720 private school charities (11%) in our data. These include charities such as Dulwich College, Dulwich Preparatory Schools Trust, James Allen’s Girls’ Schools, and St Paul’s Girls’ School. The association with the think tanks is also strong; 16 of the 59 think tanks in our data (27%) have had a member of the corporate elite on their board. They include prominent right‐wing think tanks such as Policy Exchange and the anti‐climate action organisation, The Global Warming Policy Institute, as well as some progressive organisations such as the Green Alliance. We also see a strong and statistically significant association with charities with a royal patron, and those involved in high culture—both suggesting status‐seeking social legitimation. Examples here include The Royal Opera House Covent Garden Foundation, The Royal National Theatre, the English National Ballet and The English National Opera.

Another notable finding is the tendency to join the boards of large charities; 77% of the corporate elite appointments are in the upper third of charities by income. Also of note is a tendency to join charities with global operations. The corporate elite are also somewhat more likely to be involved in organisations working on the environment, conservation and heritage, and in organisations involved in arts, culture, heritage or science. This is also suggestive of the sorts of status‐seeking and social legitimation we aim to capture with our royal patronage and high culture variables, and/or a more traditional or conservative orientation. The association with armed forces charities is also worth noting for this reason—these include, for example, the Forces in Mind Trust, the Royal Navy and Marines Charity, the National Museum of the Royal Navy and the Royal Air Force Benevolent Fund.

The expectation that the corporate elite would, due to an interesting in promoting meritocratic ideology, be more likely to join sports‐based charities and those working with young people is not supported by the findings. The positive and statistically significant association with education could still be taken to be evidence of such, although as we shall see this does not stand up in the next stage of analysis.

Turning to the remaining binary variables, and focussing on the statistically significant results, the general picture is that the corporate elite are less associated with charitable companies providing welfare and social services, or those campaigning around social justice issues, and more associated with charities seeking to shape knowledge production. So, they are more prevalent on the boards of charitable companies involved in research and in grant provision than those involved in supporting ethnic minorities, people with disabilities or older people, or those working on issues such as poverty, human rights, equality and diversity.

### 6.2 Logistic Regression Analysis

We used logistic regression to examine the relationship between our predictor variables. We report the results for three models in Table 2. In Model 1 we include four of our five constructed variables of particular theoretical interest, with Oxbridge excluded. In Model 2 we add our three ordinal variables: charity size, age and area of operations. In Model 3, we add all the remaining variables, with the groupings retained from Table 1. For all three models we include a control variable recording in how many of our sample years, if any, an organisation did not operate. This takes into account the fact that for some charities there was less time in which it was possible for the corporate elite to join the board.

**TABLE 2:** Logistic regression predicting presence of a member of the corporate elite on the board.

	Model 1	Model 2	Model 3
Royal patronage	7.08	3.26	2.77
High culture	1.76	2.06	1.57
Private school	5.58	3.12	2.62
Think tanks	13.86	6.85	5.07
Size			
Large		7.81	7.92
Medium		1.61	1.76
Small (reference category)			
Age			
41+		0.98	0.98
21–40		0.76	0.77
0–20 (reference category)			
Area of operations			
Global		1.94	1.55
International		1.88	1.49
National		1.39	1.23
Local (reference category)			
Legitimation—traditional			
Armed forces/emergency services			1.98
Arts/culture/heritage/science			1.29
Environment/conservation/heritage			1.28
Legitimation—meritocratic			
Amateur sport			0.96
Education/training			0.92
Young people			1.14
Other Charity Commission variables			
Accommodation/housing			0.91
Advocacy/advice/info			0.81
Animals			0.44
Disability			0.90
Economic/community development/employment			0.93
Ethnic group			0.82
Health/saving lives			0.93
Human rights/equality/diversity			0.62
Makes grants			1.99
Older people			0.78
Overseas aid			0.94
Poverty			1.09
Public/mankind			1.07
Recreation			1.00
Religion			0.30
Research			1.60
Years not active (control)	0.89	0.91	0.89
Pseudo R2	0.05	0.12	0.15

*N* = 31,666.

From the odds ratios in Model 1 we see that the corporate elite are almost twice as likely to join an organisation involved in ‘high culture’, compared with other charities, and more than seven times more likely to join an organisation with a royal patron compared to those without. The association with think tanks remains striking, as is the association with private schools. In Model 2 we see again the tendency of the corporate elites to join the large charities, as well as a less marked tendency to join organisations with national, international or global operations (compared to local charities). Introducing these variables reduces the associations with private school charities, those with a royal patron, and think tanks, since these organisations tend to be larger. The association with high culture, meanwhile, increases slightly, since these charities tend to be smaller.

In Model 3, the positive association with our main variables remain, although the associations are again reduced somewhat by the introduction of the other variables. In the case of the private school charities, this is largely due to the fact that 30% of these organisations are involved in grant provision, whilst in the case of think tanks it is primarily because of their involvement in research, which is also a more general positive association across the data. Education also plays a role in both, but what is most of note is the extent to which the association with education in the bivariate analysis, which anyway was not strong, reduces once involvement in private schooling is taken into account. More generally the overall picture outlined above stands up.

What does this tell us about corporate elite engagement in civil society? Whilst some existing research would lead us to expect an association with charities campaigning on social justice issues, or providing support to disadvantaged groups, with corporations and corporate elites thereby hoping to enhance their public reputation (Joel and Kyeremeh 2020), the findings align much more with sociological perspectives that emphasise class interests, social power, status and cultural legitimation as the driving forces behind extra‐corporate roles. To be clear, the results are consistent with corporate elite support for social justice issues and for welfare and social services. But in aggregative the patterns of civil society engagement are clearly more traditional and instrumental than reputational or social. As discussed, they are also more traditional than meritocratic, although as noted above the latter is more difficult to capture with the available data. There is one sense in which the corporate elite appear less traditional, and that is the association with religious organisations, which is the strongest negative association in our data, and is significant at the 1% level. As can be seen from Table 1, there are a number of religious charities with a corporate elite on the board. These are mainly Christian. They include, for example, Alpha International, the Catholic Union Charitable Trust, St Paul's Theological Centre and Tearfund. Overall, though, the patterns of corporate elite engagement are more secular than religious considering the number of charitable companies undertaking religious activities, and this holds in our regression models.

## 7 Conclusion, Limitations and Future Research

The article researches a hard‐to‐reach group, the UK corporate elite, by combining two sources of administrative data—the Charity Commission and Companies House—in a way that allows civil society appointments to be analysed systematically, over time and at scale. Making use of extensive structured data on the nature and operations of charities, as well as data from several other sources, we are able to identify which types of organisations are more likely to attract members of the corporate elite to their boards. Our findings confirm most of our hypotheses. The UK corporate elite are more likely to join the boards of larger, high‐status charities (Maclean et al. 2021), and those that support traditional upper‐class culture and class reproduction (Fisher 1983). We also find they are more likely to join organisations that seek to shape politics and society—such as foundations distributing grants, or think tanks undertaking public policy research and advocacy—than those involved in the provision of welfare and social services. Insofar as civil society engagement may play a role in legitimising social inequalities, there is stronger evidence for ‘high culture’ as a means to do so (Reeves 2019; Levine 1990), than support for meritocratic ideology (Littler 2017). Taken together, the findings are suggestive of a status‐seeking, culturally highbrow and secular economic elite, that is more traditional than meritocratic, and more concerned with shaping policy and supporting the institutions of their class, than directly supporting disadvantaged groups.

There are some limitations to our use of administrative data. We remain to some extent limited by our focus on a subset of civil society organisations, and by variables that imperfectly measure what we want to capture; and as with any study of this type we are not able to uncover the causal mechanisms giving rise to the associations we report. One notable issue is that we cannot account for the activities of the charitable companies that may encourage a member of the corporate elite to join their board. These organisations have an interest in appointing an affluent and well‐connected person to their board, and so there may be ‘pull’ factors we do not capture. It may be possible that additional data would allow for this shortcoming to be addressed, but in all likelihood qualitative methods would be the only way to put flesh on the bones of our analysis here.

That said, there is considerable scope for building on this work with further quantitative investigation. Our research is limited to charities registered in England and Wales and, data allowing, it would be interesting to examine to what extent other countries exhibit similar or distinct patterns of elite‐civil society engagement. Examining Scotland and Northern Ireland charities may also uncover differences within the UK. Finally, one important aspect we don't cover here, but plan to in future work, is how civil society engagement varies among the corporate elite, for example by gender, ethnicity, nationality, career stage, industrial sectors, or centrality in the UK intercorporate network. This will allow us to disaggregate the corporate elite and thereby to better understand the drivers of elite civil society engagement.

## Data Availability

The data that support the findings of this study are openly available in GitHub at https://github.com/Narzanin/corporate‐elites‐charities.

## Endnotes

^1^
We recognise there are important distinctions between elite and corporate philanthropy, Corporate Social Responsibility (CSR) and ‘philanthrocapitalism’ but discussing these differences is not the focus of our study. Our paper is less concerned with understanding the nature of corporate elite ‘giving’ and is instead focused on what the characteristics of charities that corporate elites engage with tell us about the social values of these economic elites.

^2^
One issue here is the direction of causation for our charity size variable since the presence of a member of the corporate elite on the board may lead to an increase in income. However, the data we have available does not suggest such an effect, and it would likely be negligible anyway given that we use an ordinal rather than a continuous measure of charity size.

^3^

https://github.com/Narzanin/corporate‐elites‐charities/tree/main.

## Appendix A Populations, Samples and Data Quality

### Charitable Companies

As noted in the main text, we examine a subset of charities which in addition to being registered at the Charity Commission of England and Wales are also incorporated under the Companies Act 2006 and therefore registered at Companies House. We utilise both data sources and use these dual registration organisations to analyse one significant mode of civil society engagement among the corporate elite.

We refer to these organisations as charitable companies, although it is worth noting that the Charity Commission itself uses this same term to refer to a narrower subset of charities. Our definition includes charities classified as charitable companies by the Charity Commission, as well as some other less common charity types in the Charity Commission data also registered at Companies House, including ‘charitable incorporated organisations’, which charitable companies are set to convert to following the introduction of new legislation, some incorporated trusts and a miscellaneous ‘Other’ category.

For the purposes of this research, the key difference between charitable companies (as we define them) and unincorporated charities (i.e. those not registered at Companies House) is that in the case of charitable companies we can definitively link the organisation's trustees with members of the corporate elite over a 10 year period. The substantive difference between these two sets of charities, meanwhile, lies in their legal structures and the liabilities of their trustees. A charitable company is legally considered a separate entity from its trustees, similarly to a commercial company. This structure allows the charity to do things in its own name, such as employing staff, entering into contracts, or owning property. In contrast, an unincorporated charity, often structured as a trust or an unincorporated association, does not provide the same separation, and the trustees are more likely to be personally liable. This type of charity cannot enter into contracts or control property in its own name; such actions must be handled by the trustees on behalf of the charity. Charitable companies also adhere to stricter registration and reporting requirements, meaning financial reporting for these charities is more extensive.

The charitable companies in our analysis are identified from Charity Commission data on the basis of having recorded a valid company registration number in the relevant field of the Charity Commission's datafile. Reported company numbers were verified using the Companies House API and a total of 207 organisations were found to have provided an invalid number. In some cases this was a result of the organisation reporting another official identification number, such as the Charity Commission's own organisation number or charity number. In other cases it was due to a basic error, such as a single character in the ID number being wrongly transcribed or missing. In other cases, we were not able to determine the reason for the erroneous company number.

For each of these 207 organisations we searched the Companies House API using the organisation name appearing in the Charity Commission data. In the event that an exact match was returned (78 cases) we replaced the reported company registration number with the company registration number associated with the organisation name in Companies House records. For the remaining 129 organisations we manually checked Charity Commission and Companies House records to attempt to match the charity to an incorporated entity. We were thereby able to identify a further 19 valid company registration numbers. The remaining organisations were dropped from our data since it is not possible using our method to verify whether a corporate elite has been appointed to the board. We also dropped 337 charitable companies which had not reported any financial data at the time of data extraction, and 342 that were registered after 31 December 2022.

We were then left with a total of 31,666 charities registered before the end of our time period, reporting a valid company registration number, and at least one year of financial data at the time of data extraction (4 January 2024); these charities make up 98.62% of the potential cases (those charities reporting a company registration number) in the Charity Commission datafile.

The charitable companies included in our analysis make up approximately 17.7% of the total population of charities active at the time of data extraction (N = 178,413). In Table A1 we compare the charitable companies in our main data with 126,858 unincorporated companies for which we were able to construct an equivalent dataset with the same method. The original Charity Commission datafile contained a total of 146,304 unincorporated charities, and the greater loss of data in constructing this equivalent dataset reflects the less comprehensive coverage for this type of organisation.

**TABLE A1:** Count, percentage for unincorporated charities and charitable companies by predictor variables.

		Unincorporated charities	Charitable companies
		*N* (%)	*N* (%)
Royal patronage	Yes	285 (0.22)	404 (1.28)
	No	126,573 (99.78)	31,262 (98.72)
High culture	Yes	1618 (1.28)	960 (3.03)
	No	125,240 (98.72)	30,706 (96.97)
Private school	Yes	672 (0.53)	720 (2.27)
	No	126,186 (99.47)	30,946 (97.73)
Oxbridge	Yes	96 (0.08)	32 (0.1)
	No	126,762 (99.92)	31,634 (99.9)
Think tank	Yes	7 (0.01)	59 (0.19)
	No	126,851 (99.99)	31,607 (99.81)
Size	Large	42,285 (33.33)	10,580 (33.41)
	Medium	42,284 (33.33)	10,499 (33.16)
	Small	42,289 (33.34)	10,587 (33.43)
Age	41+	31,738 (25.02)	2680 (8.46)
	21–40	36,995 (29.16)	8196 (25.88)
	0–20	58,125 (45.82)	20,790 (65.65)
Area of operations	Global	3654 (2.88)	1995 (6.3)
	International	9493 (7.48)	2797 (8.83)
	National	15,793 (12.45)	5464 (17.26)
	Local	97,918 (77.19)	21,410 (67.61)
Legitimation—traditional			
Armed forces/emergency services	Yes	801 (0.63)	187 (0.59)
	No	126,057 (99.37)	31,479 (99.41)
Arts/culture/heritage/science	Yes	21,767 (17.16)	7088 (22.38)
	No	105,091 (82.84)	24,578 (77.62)
Environment/conservation/heritage	Yes	13,647 (10.76)	4590 (14.5)
	No	113,211 (89.24)	27,076 (85.5)
Legitimation—meritocratic			
Amateur sport	Yes	21,252 (16.75)	4105 (12.96)
	No	105,606 (83.25)	27,561 (87.04)
Education/training	Yes	60,750 (47.89)	11,258 (35.55)
	No	66,108 (52.11)	20,408 (64.45)
Young people	Yes	72,167 (56.89)	17,242 (54.45)
	No	54,691 (43.11)	14,424 (45.55)
Other charity commission variables			
Accommodation/housing	Yes	5317 (4.19)	2580 (8.15)
	No	121,541 (95.81)	29,086 (91.85)
Advocacy/advice/info	Yes	27,193 (21.44)	14,271 (45.07)
	No	99,665 (78.56)	17,395 (54.93)
Animals	Yes	3391 (2.67)	839 (2.65)
	No	123,467 (97.33)	30,827 (97.35)
Disability	Yes	36,251 (28.58)	11,255 (35.54)
	No	90,607 (71.42)	20,411 (64.46)
Economic/community development/employment	Yes	13,369 (10.54)	7007 (22.13)
	No	113,489 (89.46)	24,659 (77.87)
Ethnic group	Yes	9990 (7.87)	4319 (13.64)
	No	116,868 (92.13)	27,347 (86.36)
Health/saving lives	Yes	19,177 (15.12)	7808 (24.66)
	No	107,681 (84.88)	23,858 (75.34)
Human rights/equality/diversity	Yes	4366 (3.44)	1757 (5.55)
	No	122,492 (96.56)	29,909 (94.45)
Makes grants	Yes	49,744 (39.21)	8449 (26.68)
	No	77,114 (60.79)	23,217 (73.32)
Older people	Yes	38,894 (30.66)	9317 (29.42)
	No	87,964 (69.34)	22,349 (70.58)
Overseas aid	Yes	7771 (6.13)	1963 (6.2)
	No	119,087 (93.87)	29,703 (93.8)
Poverty	Yes	23,503 (18.53)	8329 (26.3)
	No	103,355 (81.47)	23,337 (73.7)
Public/mankind	Yes	63,251 (49.86)	12,511 (39.51)
	No	63,607 (50.14)	19,155 (60.49)
Recreation	Yes	12,955 (10.21)	3084 (9.74)
	No	113,903 (89.79)	28,582 (90.26)
Religion	Yes	27,481 (21.66)	5626 (17.77)
	No	99,377 (78.34)	26,040 (82.23)
Research	Yes	7310 (5.76)	5085 (16.06)
	No	119,548 (94.24)	26,581 (83.94)

As can be seen from Table A1, there are differences between the two categories of organisations when it comes to our predictor variables. Unincorporated charities, for example, tend to be older, and somewhat narrower in terms of their geographical activities. Since our measure of charity size divides the organisations into three equal ordinal categories, it doesn't allow for comparison between the two sets of organisations, but the main difference that emerges is that incorporated charities tend to be much larger. The median gross income, on which the charity size variable is based, is £164,510 for the unincorporated charities and £13,396 for the incorporated charities.

The charitable companies used in our analysis are not a probability sample from this larger population of charities, but they are a large sample. Nevertheless, we are not able to reliably infer from our models the extent of corporate elite engagement in the charity sector since there are reasons to believe that a charity being incorporated would independently impact on the probability of a member of the corporate elite joining the board, e.g. since as noted incorporation offers greater protection for trustees. That said, there is no good reason to believe that the patterns of associations reported here would not hold for both sets of charities, and were we hypothetically able to introduce incorporation as a control variable we would be able to infer the extent of corporate elite engagement in the charity sector as a whole. Given that we are not attempting to quantify corporate elite appointments using our models, but rather to reveal associations with the predictor variables, we consider our results to be generalisable to the broader population of charities in England and Wales.

It is furthermore important to note that there are many organisations that would conventionally be considered civil society organisations, which are not registered charities, either because their activities would not fall within stipulated ‘charitable purposes’, because they wish to avoid certain restrictions that come with charitable status, such as involvement in politics, or because they have a different corporate form. These include, for example, non‐profit companies not registered as charities, which like charitable companies are conventionally registered at Companies House as a ‘company limited by guarantee’, Royal Charter companies, which are incorporated by Royal Prerogative, and unincorporated associations. Appointments to these categories of organisations are not included in our data.

### Data Quality

When registering with the Charity Commission organisations are required to provide details on what a charity does, who they help, and how this is achieved. Charities can select multiple classifications for their charity in each category. We used this data to create variables in the analysis.

There were 34 such categories in the original Charity Commission datafile. A number of these categories were too broad to be used to construct variables for analysis (e.g. ‘Other Defined Groups’, ‘General Charitable Purposes’, ‘Other Charitable Activities’), and we selected 22 variables that we considered to be theoretically useful. As noted in the main text, detailed guidance on the data provided by the Charity Commission is available online (Charity Commission 2024), and the original Charity Commission data, along with the Python scripts used to construct each of the above variables, and to produce the final dataset, are available on GitHub.

99.76% of the organisations in our data report activities in at least one of the 22 categories of activities we include in our analysis. On average, an organisation reports five different categories in our data (mean = 5.3, std = 2.9). The organisations not reporting any activities in our categories (n = 76) define their activities using very general categories that we do not include in our analysis. We do, however, have data to construct the first eight variables in our data tables for these organisations, and their non‐response on the 22 charities classifications is not treated as missing data. They are therefore included in our analysis. Excluding these organisations, however, does not impact on our findings.

Our use of this data, and our reliance on administrative data generally, raises issues around data quality. But one advantage of the particular type of data we use is that they are obtained from official bodies with legal and regulatory powers, and are therefore of a much higher quality than other sources of ‘found data’ or ‘big data’, for example commercially produced datasets and/or user generated data from digital platforms. The data are self‐reported, and of course this means some degree of human error is inevitable, but this would also be the case were we to have relied, for example, on survey data or researcher generated categories.

Finally, as noted in the main text, there are additional variables that feature in the original Charity Commission datafile which are not used in our analysis. In some cases this is because the variable is not of theoretical interest or practical use. In other cases the variable is potentially of some theoretical interest, but was not included in our analysis because of the volume of missing data. As we note in the main text, the majority of these variables are from the Charity Commission's financial datafiles. We report the counts and percentage of missing data for these variables, and others not used in our analysis, in Table A2.

**TABLE A2:** Count and percentage of missing data for Charity Commission variables not included in the analysis.

Charity commission variable	Missing data
	*N* (%)
Charity activities	100 (0.31)
Charity contact address 1	15 (0.05)
Charity contact address 2	32 (0.10)
Charity contact address 3	7196 (22.41)
Charity contact address 4	18,724 (58.31)
Charity contact address 5	27,368 (85.23)
Charity contact postcode	251 (0.78)
Charity contact phone	52 (0.16)
Charity contact email	3063 (9.54)
Charity contact web	5821 (18.13)
Charity gift aid	172 (0.54)
Charity has land	117 (0.36)
Mean government income	24,690 (76.89)
Mean count volunteers	3224 (10.04)
Charity raises funds from public	3482 (10.84)
Charity professional fundraiser	12,032 (37.47)
Charity agreement professional fundraiser	29,602 (92.19)
Charity commercial participator	11,725 (36.52)
Charity agreement commercial participator	30,235 (94.16)
Grant making is main activity	19,264 (60.00)
Charity receives govt funding contracts	3712 (11.56)
Charity receives govt funding grants	3712 (11.56)
Charity has trading subsidiary	3412 (10.63)
Trustee also director of subsidiary	26,854 (83.63)
Trustee payments acting as trustee	22,072 (68.74)
Trustee receives payments services	23,349 (72.72)
Trustee receives other benefit	23,349 (72.72)
Trustee resigned employment	3642 (11.34)
Employees salary over 60k	3685 (11.48)
Does trustee receive any benefit	3560 (11.09)

*N* = 32,109.
